# Commentary: Physiological and Psychological Impact of Face Mask Usage during the COVID-19 Pandemic

**DOI:** 10.3390/ijerph17186655

**Published:** 2020-09-12

**Authors:** Jennifer L. Scheid, Shannon P. Lupien, Gregory S. Ford, Sarah L. West

**Affiliations:** 1 Department of Health Promotion, Daemen College, Amherst, NY 14226, USA; 2 Department of Physical Therapy, Daemen College, Amherst, NY 14226, USA; gford@daemen.edu; 3 Department of Psychological Sciences, Daemen College, Amherst, NY 14226, USA; slupien@daemen.edu; 4 Department of Biology & Trent/Fleming School of Nursing, Trent University, Peterborough, ON K9L 0G2, Canada; sarahwest@trentu.ca

**Keywords:** masks, pandemic, COVID-19, face covering, physiology, basic phycological needs, exercise

## Abstract

In this commentary, we discuss the physiological effects of wearing masks for prolonged periods of time, including special considerations, such as mask wearing among those who engage in exercise training, and concerns for individuals with pre-existing chronic diseases. In healthy populations, wearing a mask does not appear to cause any harmful physiological alterations, and the potentially life-saving benefits of wearing face masks seem to outweigh the documented discomforts (e.g. headaches). However, there continues to be controversy over mask wearing in the United States, even though wearing a mask appears to have only minor physiological drawbacks. While there are minimal physiological impacts on wearing a mask, theoretical evidence suggests that there may be consequential psychological impacts of mask wearing on the basic psychological needs of competence, autonomy, and relatedness. These psychological impacts may contribute to the controversy associated with wearing masks during the COVID-19 pandemic in the United States. After we discuss the physiological impacts of mask wearing, we will discuss psychological effects associated with wearing masks during the COVID-19 pandemic.

## 1. Introduction

On March 11, 2020, the World Health Organization declared COVID-19 to be a global pandemic [1]. At the time of the announcement, the global number of COVID-19 cases was increasing daily, and five months later, worldwide cases continued to increase [2]. Many countries, including the United States, were on a pause, entered a lockdown, or sheltered in place. As such, many communities were instructed to stay in their home except to obtain essential supplies. Individuals, such as healthcare providers, grocery store clerks, and delivery personnel, were considered essential employees and continued with their jobs, but many individuals began working from home for an extended period of time. With the severity of the global pandemic increasing, on April 3, 2020, the Centers for Disease Control and Prevention (CDC) in the United States recommended that individuals wear a face mask in public if they cannot distance at least six feet from others, to help prevent the transmission of COVID-19 [3,4]. 

This recommendation (and in some cases, local and/or state ordered mandates) for mask use seems to have created controversy among the general public, especially in the United States. In addition, workers in many professions not previously accustomed to mask use were suddenly expected to work while wearing masks. This includes grocery store and foodservice workers, bartenders, teachers, childcare providers and laborers, among others. This has led to numerous concerns, with masks being perceived as uncomfortable, cumbersome, a nuisance, or inconvenient. It has even resulted in worries that extended mask use might be unhealthy or dangerous. In this commentary, we will discuss the varying types of masks available for use, and then explore the physiological effects of wearing masks for prolonged periods of time, including special considerations, such as mask wearing among those who engage in exercise training, and concerns for individuals with pre-existing chronic disease. We will also discuss the psychological impact of wearing masks during the COVID-19 pandemic, and how this may relate to individuals’ perceptions of, and willingness to, comply with mask wearing.

## 2. Types of Masks

To begin, it is important to understand the different types of masks available and what they are designed to do. Medical-grade masks, which include surgical and N95 respirator masks, are primarily designed to protect the wearer from airborne particles and droplets, as well as from bodily or other hazardous fluids. Surgical masks and N95 respirators are both regulated in the USA by the U.S Food and Drug Administration (FDA). The FDA describes a surgical mask as a “loose-fitting, disposable device that creates a physical barrier between the mouth and nose of the wearer and potential contaminants in the immediate environment,” and an N95 respirator as a “respiratory protective device designed to achieve a very close facial fit and very efficient filtration of airborne particles” [5]. A systematic review and meta-analysis by Smith and colleagues found no significant difference between N95 and surgical masks in associated risks of respiratory infection in healthcare workers, although N95 respirators were reported to have less filter penetration and less face-seal leakage when compared to surgical masks [6]. Medical workers can sometimes be seen wearing face shields. A face shield is typically used (in combination with a mask) because it protects the wearers’ eyes from exposure from respiratory droplets of an infected person. The CDC “does not currently recommend use of face shields as a substitute for masks” [4]. Non-medical cloth masks, sometimes called face coverings, on the other hand, can be created using a variety of materials or a combination of materials, such as chiffon, cotton, flannel, silk, or other synthetics, and are designed to decrease the transmission of respiratory droplets during breathing, speaking, or coughing [7]. In the United States, as of July 2020, the CDC recommends “that people wear cloth face coverings in public settings when around people outside of their household, especially when other social distancing measures are difficult to maintain” [4]. Contrary to what some may believe, these cloth masks/face coverings are not designed to protect the mask wearer, but rather, they are intended to protect people within close proximity of the wearer [8].

Because we have not experienced a time in recent history that has mandated the use of cloth face coverings in public spaces, the majority of research exploring the impact of masks on human physiology has been conducted on surgical masks [9] and N95 respirators [10,11,12]. For example, a recent animal model study suggested that non-contact transmission of SARS-CoV-2 (the virus responsible for COVID-19) could be reduced from ~67% (no masks) to ~17% when surgical masks are worn [13]. However, preliminary studies do suggest that cloth masks are important in reducing the spread of COVID-19 [7,14,15]. A recent study of COVID-19 in San Francisco suggested that the most effective public interventions in reducing virial spread were sheltering in place and contact tracing efforts. However, once shelter in place procedures were lifted, adopting the use of cloth masks by the community was deemed an important and effective means of reducing virus spread [8]. Chu and colleagues’ recent meta-analysis of 172 observational studies examined the impact of face masks and physical distancing on the transmission of SARS-CoV-2, and reported that face masks (in addition to physical distancing) decreased the transmission of the virus [16]. Thus, growing evidence is beginning to demonstrate that there are important benefits to wearing masks, which in turn, calls for the need to examine whether or not there are important drawbacks. Although there are some differences between cloth masks and medical-grade masks for protection against airborne pathogens that can be transmitted in aerosol form, like SARS-CoV-2 [7,14], surgical masks and the N95 respirators can be used as models to examine any physiological adaptions that occur while wearing a mask for an extended period of time.

## 3. Physiology of Prolonged Mask Use

### 3.1. Physiological Need: Respiration

We understand that masks, both medical grade and cloth, can be effective in reducing virus transmission. However, when dissecting whether there is a physiological basis behind the mask-wearing controversy, the first question that should be examined is: are there any changes to respiration while wearing a mask? In human physiology, there are two types of respiration: internal and external respiration. Internal respiration is the process responsible for oxygen consumption that occurs at the cell, while external respiration is the process of transporting oxygen from outside the body to the cell that needs the oxygen (and concurrently transporting carbon dioxide from the cell to the atmosphere). Scientists and clinicians can measure lung function through a variety of outcomes, such as tidal volume (the lung volume displaced from normal inhalation to normal exhalation), frequency of breaths (respiratory rate, or number of breaths per minute), and total ventilation (the product of the tidal volume and the breathing rate). Roberge and colleagues [11] investigated the impact of wearing an N95 respirator in healthy adult healthcare workers, while walking slowly on a treadmill (1.7 miles/h and 2.5 miles/h) for a 1-hour period. One hour of slowly walking while wearing an N95 respirator did not impact respiration; specifically, there was no impact on the respiratory rate, tidal volume, or the total ventilation (total air flow per minute) [11]. This study suggests that N95 respirators do not change breathing patterns during low intensity physical activity, (i.e. light walking, which is a typical occupational activity at most hospitals or other places of work). Longer simulations have also been conducted in a laboratory setting. For example, during a four-hour computer simulation using three different classes of N95 respirators, Roberge and colleagues [17] found a 3% increase in inhalation and exhalation resistance, which was likely caused by exhaled moisture that was retained by the mask. This increased resistance means that greater air force would be needed for the air to pass through the mask, which in humans, could mean an increase in respiratory muscle use. However, the authors concluded that these changes were relatively minor, and it is unlikely that the individual wearing the mask would be able to perceive these changes in breathing [17]. These studies [11,17] demonstrate that there may be a small increase in breathing resistance while wearing a mask, but it does not seem to impact tidal volume or the frequency of breaths. Additionally, the reported changes in breathing resistance are small enough to have minimal clinical implications. Presumably, cloth masks that are less tight fitting than N95 masks will have even less of an impact on breathing resistance. 

### 3.2. Physiological Need: Oxygen Delivery

If respiration is essentially unaffected by mask use, the second physiological question to examine is: are there any changes to oxygen delivery in the blood while wearing a mask? The majority of oxygen (98%) is carried through the bloodstream by hemoglobin. The oxygen-dissociation curve represents a very important mechanism of human physiology involved in loading oxygen onto hemoglobin at the lungs, and offloading oxygen from hemoglobin at tissues that require oxygen delivery. The oxygen-dissociation curve is relatively flat at the top, meaning that, even if there are changes in the partial pressure of oxygen in the lungs, those changes are rarely reflected in the arterial oxygen concentrations. The body is very efficient at maintaining oxygen saturation around 98%, with normal ranges between 90–98% arterial hemoglobin saturation. Low partial pressures of oxygen are found in tissues around the body, and the steep curve helps with off-loading oxygen, meaning that, even if there are small changes in the partial pressure of oxygen at the tissue, the oxygen is off loaded from the hemoglobin to be delivered to the active tissue [18].

Tightly regulated oxygen levels are reflected in research that examines the impact of wearing masks on oxygen saturation levels (i.e., hemoglobin saturation levels). For example, an observational study of 52 surgeons wearing surgical masks revealed a decrease in arterial O_2_ saturation from approximately 98% before surgery to 96% after surgery, which ranged from 1–4 h in length. Additionally, an increase in heart rate from ~85 bpm before surgery to 90 bpm after surgery was also noted [9]. While these changes in O_2_ saturation and heart rate were statistically significant, they are not clinically important, given that post-surgery O_2_ saturation numbers remained in the normal range (90–98%) [9], as did the heart rate values (normal resting: 60–100 beats per min) [18]. 

In addition to blood oxygen levels being tightly regulated, carbon dioxide (CO_2_) levels are also tightly controlled. If CO_2_ concentrations begin to rise in the blood, a number of physiological mechanisms occur to decrease the CO_2_, including greater ventilation to increase gas exchange at the lungs. In 2013, Rebmann and colleagues [10] examined the impact of N95 respirators alone compared to N95 respirators with a surgical mask overlay in nurses during a 12-h shift. Transcutaneous CO_2_ became elevated during the 12-h shift, both in nurses wearing an N95 respirator and N95 respirator with a surgical mask overlay [10]. However, while CO_2_ elevations were statistically significant after the 12-h shift, like the O_2_ saturation data discussed above, the changes are likely not clinically important, as CO_2_ remained within healthy normal ranges (< 45 mmHg) [10]. Additionally, there were no changes in blood oxygen concentrations or blood pressure during the 12-h shifts [10]. These studies [9,10] support that wearing a medical-grade mask does not appear to impact blood oxygen or carbon dioxide concentrations.

## 4. Discomforts

While there may be minimal concern regarding physiological changes in respiration and/or to O_2_ saturation or CO_2_ levels while wearing a mask, it is possible that there could be greater discomforts during prolonged periods of use. Prolonged use (≥12 h) of masks (N95 respirator or N95 respirator with a surgical mask overlay) has been associated with complaints of headaches, light-headedness, as well as an increase in perceived exertion and perceived shortness of breath [10], even though the data discussed above supports no actual oxygen shortage. Headaches are also a prominent symptom reported by healthcare workers wearing masks during the COVID-19 pandemic [19]. A survey of 158 healthcare workers in Singapore found that 81% experienced headaches while wearing N95 respirators and protective eyewear for an average of 6 h per day [19]. Headaches were most likely to develop in people who wore both masks and protective eyewear for more than four h and had pre-existing headaches [19]. Lim and colleagues [20] reported an increased proportion of headaches (37%) among 212 healthcare works during the Severe Acute Respiratory Syndrome (SARS) outbreak in 2003, involving a virus similar to SARS-CoV-2 that impacts the upper respiratory tract. Thirty-three percent of the workers had more than six headaches in one month [20]. Similar to the findings of Ong and colleagues [19], Lim and colleagues [20] demonstrated that headaches were most likely to develop in people who wore masks for longer than 4 h and had pre-existing headaches.

Many factors may contribute to the increased number of new headaches while wearing a mask, including the design of masks which rely on tight elastic straps [19]. Elastic straps in combination with a tight fit may result in face pain behind the ears or other contact points [19]. Indirect factors may also contribute to headaches while wearing masks, for example, inadequate hydration and irregular eating patterns [19]. Additionally, unrelated factors to wearing masks, such a sleep deprivation, and physical and emotional stress [19], are also thought to possibly contribute to headaches, especially during pandemic situations. However, based on the data previously discussed, we believe it unlikely that headaches are caused by physiological changes to O_2_ and CO_2_ balance.

In addition to headaches, other discomforts have been reported while wearing a mask, including acne [21], nasal bridge scarring [22], facial itching [22], rash/irritation [23] and discomfort related to increased facial temperatures [12,24]. N95 respirators that were worn for one hour increased skin temperature from normal levels to above 34.5 °C; however, while this did lead to discomfort, facial skin temperature decreased quickly following the removal of the respirators [12]. This study was corroborated using thermal imagery by Luximon and colleagues [25] when masks were only worn for five minutes, with N95 masks increasing skin temperature more than surgical masks. Subjects reported increased humidity, breathing difficulty, and overall discomfort when speaking with a mask on, compared to being silent. Thus, while masks do not appear to cause harmful physiological alterations, they do appear to cause some discomfort, including headaches and face irritation. Because most individuals are not required to wear the tight fitting N95 medical-grade masks, loose cloth-type masks should not restrict breathing outcomes or result in the same level of discomfort as reported among those who wear medical-grade masks. 

## 5. Special Considerations

### 5.1. Exercising

Although moderate physical activity (walking on a treadmill at 2.5 miles/h) while wearing an N95 respirator did not appear to have any effect on ventilation [11], to date, there are not many studies that have examined higher intensities of exercise while wearing a mask. The World Health Organization does not currently recommend wearing a mask while exercising [26], and the CDC recognizes that it may be difficult to wear a mask during high intensity physical activities [4]. Research examining military grade respirators suggest that there may be alterations to ventilation during high exercise intensities above 85% of maximum oxygen consumption [27]. This finding was also supported by a recent study that examined the impact of wearing a surgical vs. N95 face mask on cardiopulmonary exercise capacity in 12 healthy males. It was reported that during an ergometer incremental exertion test (i.e., high intensity test to exhaustion), pulmonary function and ventilation were significantly reduced with the use of either mask. Cardiopulmonary exercise capacity was also reduced with mask wearing (lower peak blood lactate response), and participants also reported discomfort while wearing the mask, especially the N95 [28]. It is important to note, however, that these studies examined very high intensity exercise, a level likely higher than an average workout for most individuals. Humidity [29], temperature [29], type of mask [28] and intensity of exercise [28] all appear to impact the effects of a mask during exercise, and should be taken into consideration if making a choice to wear a mask during exercise or not. To our knowledge, there are no studies that examine O_2_ saturation or the partial pressure of oxygen in response to exercise with a mask. At these high exercise intensities, we would expect that, while oxygen and carbon dioxide concentrations in the blood may remain relatively stable, there would be discomfort related to the temperature of the skin created by the mask and the breathing resistance caused by the mask. Exercisers may either need to persist through increased discomfort or lower their exercise intensity while wearing a mask if discomfort exists. Additionally, wearing loser cloth masks made with wicking materials that do not hold moisture should improve comfort during exercise. 

### 5.2. Special Populations

Children, pregnant women, and people with chronic diseases (specifically respiratory illnesses) are also important populations to consider during the current wide-spread recommendation for mask use. In response to the COVID-19 pandemic, the CDC currently recommends that children over the age of two years wear a cloth mask [4]. To date, there is no published research examining the physiological impact of children wearing cloth masks; however, Smart and colleagues [30] examined children wearing facemasks for the purpose of protection against air pollution. Similar to studies in adults [12], the children had discomfort related to increased facial temperatures [30]. The children had the most discomfort (i.e., faces becoming too hot) when they were performing activities such as running [30]. In pregnant women, a recent systematic review demonstrated that N95 masks worn for a short period of time do not impact maternal heart rate, fetal heart rate, respiratory rate, or blood oxygen concentration levels [31]. While prolonged use of cloth masks in pregnant women has not yet been studied, short term use of masks in pregnant women does not appear to create any physiological aberrations. 

According to the CDC, “cloth face coverings should not be placed on … anyone who has trouble breathing, or is unconscious, incapacitated, or otherwise unable to remove the mask without assistance” [4]. However, each state in the United States has further guidelines regarding the use of face coverings. That said, there is no specific list of chronic diseases that may be exempt from wearing masks. Some studies have explored the use of medical grade masks in various chronic diseases. For example, one study examined the impact of wearing an N95 mask on physiological outcomes in 39 patients with end-stage renal disease on hemodialysis during the 2003 SARS outbreak [32]. Each patient wore a new N95 mask for 4 h during their dialysis session. Seventy percent of patients showed a reduction in partial pressure of oxygen, and about 19% developed hypoxemia (i.e., low levels of oxygen in the blood). As well, the respiratory rate increased, and there was an increase in the report of chest discomfort and respiratory distress in patients. Therefore, in this particular study, the use of an N95 mask was associated with adverse outcomes in patients with end-stage renal disease [32].

Another study examined the impact of wearing either a half-mask respirator or an N95 mask among individuals with mild pulmonary diseases, including asthma, chronic rhinitis, and chronic obstructive pulmonary disease (COPD). Participants completed a series of simulated work tasks, both sedentary and more active tasks, such as driving, walking across a room, and stocking shelves with groceries, while wearing one of the masks. The half-mask respirator resulted in prolonged inspiration and a compressed expiratory phase, and results indicated that individuals with asthma may have a particularly difficult time adapting to this type of mask [33]. The Asthma and Allergy Foundation of America recently published an article in response to the COVID-19 mask and face covering recommendation, and highlights that, for people with mild and/or well-controlled asthma, wearing a mask is likely not an issue. However, for those who have severe disease and have frequent issues related to their asthma, wearing a mask may pose a risk [34].

Based on the wide range of chronic diseases and severity of each person’s disease, the decision to wear a mask will likely need to be made individually and with consultation from a physician, given the individual’s particular circumstances. Individuals with pre-existing chronic diseases, such as diabetes, hypertension and obesity (metabolic syndrome) are at an increased risk of hospitalization and have increased mortality with COVID-19 [35]. Therefore, the importance of wearing masks is underscored to help protect this vulnerable population. That said, if an individual with a chronic disease is unable to safely wear a mask, the responsibility may fall to the otherwise healthy individuals to ensure that they are wearing masks to protect the vulnerable. 

Thus, it seems that the potentially life-saving benefits of wearing face masks would largely outweigh the discomforts for most individuals. So why is there so much wide-spread resistance to wearing masks, when doing so seems to have only minor physiological drawbacks? The answers might lie in the psychological impact of wearing a mask during the COVID-19 pandemic. Specifically, theoretical evidence suggests that mask wearing may have important implications for meeting basic psychological needs, such as competence, autonomy, and relatedness.

## 6. Psychological Impacts of Wearing a Mask

### 6.1. Psychological Need: Autonomy

Human behavior often stems from subjective experience, which directly relates to how individuals interpret events in their world and how those events relate to meeting their basic, fundamental needs. Self-determination theory [36] delineates three universal, fundamental needs for optimal wellbeing: autonomy, psychological relatedness, and competence. Fulfilling these psychological needs may be at the heart of mask-wearing attitudes and compliance. Autonomy, the ability to have free will and choice over one’s actions, is considered one of these basic psychological needs. When these feelings of autonomy and personal freedom are taken away, people often experience psychological reactance [37,38], which can lead to a number of negative responses. Specifically, reactance often occurs when people perceive a threat to their freedom of choice, which in turn, leads to efforts to restore that freedom, such as non-compliance [39], anger [40], and derogation of the source [41], etc. The recommendations for wearing masks in public, and in some cases mandates, may impact perceptions of autonomy if people feel like they do not have a choice over whether or not to wear them. 

Furthermore, regulations and mandates related to mask wearing in the United States came without public input or voting, and often times without any real ability to enforce mask-wearing behavior or sanction those who do not comply. This exact condition, imposed guidelines without enforcement, has been shown to lead to the lowest amount of compliance as well as lower perceived legitimacy of the policy itself [42]. It is possible that these same attitudes and non-compliant behaviors could be elicited if there were mandates against wearing masks, and people felt like they did not have the freedom to choose to wear face coverings for protection during the COVID-19 pandemic. Thus, it may be the case that recommendations and mandates to wear masks lead to diminished feelings of autonomy. This in turn may be partially what is leading to negative attitudes towards mask wearing versus stemming from any real physiological effects.

### 6.2. Psychological Need: Relatedness

Negative attitudes and non-compliance towards mask wearing are likely enhanced, given that the recommendations and mandates have become so polarized. Towards the beginning of the COVID-19 pandemic, and shortly after the CDC released its recommendations for mask wearing in public, Democrats and Republicans differed somewhat in their reports of whether or not they always wore a mask in public (38% and 24%, respectively), according to an Axios/Ipsos poll [43]. This percentage increased over time among both groups as the severity of the pandemic increased. However, mask wearing likely became a more polarized political issue when the CDC face mask recommendations were dismissed (both in word and action) by major government figures. This, in turn, may have enhanced an ‘us’ versus ‘them’ feeling between members of each party, and may explain why, over time, there became an even greater divide in mask wearing among Democrats (65%) and Republicans (35%) a short two months later [43]. 

This is relevant for another of the three basic psychological needs—relatedness, which refers to feeling socially connected to others [36]. This is very similar to the need to belong, which is referred to as a desire for interpersonal attachment, and has also been shown to be a fundamental human need [44]. Because of these motivations to connect, humans may search for belonging and relatedness among those perceived to be part of their own ingroup, and thus are more likely to conform to various norms of the group as a way to fit in [44,45]. For example, a study examining behavior change following a respiratory etiquette intervention during flu season indicated that participants were more comfortable wearing a mask if they perceived it to be socially acceptable [46]. 

Ingroups and feelings of ‘us’ versus ‘them’ can be created in the most trivial of circumstances, and just the mere division into separate groups can be enough to trigger in-group favoritism and out-group discrimination [45]. This in-group favoritism is enhanced when the ingroup is a strong part of one’s identity [47], and if individuals feel that the value of their ingroup is being threatened [48], which is likely a reality of the current polarized political system. Therefore, one’s political party, especially if the party is a meaningful part of one’s identity, may provide an important means to fulfill the need to belong, and thus can lead to strong party favoritism and conformity to group norms. Thus, mask adherence may, in part, be a function of whether or not mask wearing is perceived to be normative among one’s political party. Specifically, research on affective responses to mask wearing during the COVID-19 pandemic has shown that how strongly one identifies as a Democrat or Republican corresponds to their level of positivity and negativity towards mask wearing [49]. 

In addition to one’s partisanship, the gender norms people subscribe to may also be related to mask wearing. For example, there are certainly social pressures in place for men to be tough and not appear weak (e.g., [50]), which has been thought to contribute to men engaging in riskier health-related behaviors [51]. Research related to mask wearing during previous pandemics has indicated that men are less likely to wear masks than women (both during the H1N1 [52,53] and SARS [54] pandemics). Similarly, mask-wearing research during the COVID-19 pandemic indicates that men who more strongly endorse the belief that men should be tough, were more likely to have negative feelings and less likely to have positive feelings towards wearing a mask [49]. Another study examining mask wearing during the COVID-19 pandemic suggests that the negative feelings that men have towards masks may predict actual intentions to wear a mask in public. Specifically, men’s lack of intention to wear a face mask was mediated by negative feelings while wearing a mask (i.e., that it is shameful, a sign of weakness, or not cool) [55]. These studies may suggest that finding relatedness among their male ingroup corresponds to less favorable mask-wearing attitudes and intentions. Thus, given the evidence above, it is possible that the psychological need to relate to others may be, in part, leading to negative attitudes towards wearing masks, rather than any real physiological effects or discomforts.

### 6.3. Psychological Need: Competence

Conforming to group norms can provide an important means to fulfill belongingness needs, but conformity is also a way for people to gain appropriate information on what is correct behavior in a given situation. In other words, people may look to those around them for information on what they should be doing [56], such as whether or not they should be wearing masks [55]. This is enhanced during situations of uncertainty and crisis, and there seems to be a lot of that to go around during the COVID-19 pandemic. For example, it was initially very unclear whether or not masks were effective and should be worn. The U.S. Surgeon General indicated that masks were, “NOT effective in preventing the general public from catching Coronavirus” [57], and the CDC did not recommend face masks for people who were well [58]. However, a month later, both reversed their position, indicating that masks should be worn [4,59]. In addition to the lack of clarity concerning mask effectiveness, information about whether or not the general public should wear masks even if they were effective was unclear because of supply-chain issues. In fact, some governmental figures indicated that the general public should not buy masks because “if healthcare providers can’t get them to care for sick patients, it puts them and our communities at risk” [57], which likely further complicated the messages about whether or not masks should be worn. In fact, Dr. Anthony Fauci, the leading infectious disease expert in the U.S., stated in a news interview that not recommending masks in the beginning so that they could be available for healthcare workers was “detrimental in getting the message across” [60]. Furthermore, research investigating the Ebola epidemic of 2014 has shown that, when public health-related messages are communicated among the general public (e.g., on social media platforms), they are more likely to include misinformation if the messages also contain political content. Thus, the politicization of disease outbreaks can serve to spread misinformation [61], which is likely occurring during the COVID-19 pandemic. Further adding to the confusion, COVID-19 is a novel coronavirus that we do not know much about.

All of these mixed messages, misinformation, and lack of medical and scientific knowledge about COVID-19 create a lot of uncertainty and threatens the ability to satisfy the last of the three basic psychological needs—competence, which refers to feeling like we are effective, capable, and have mastery over our circumstances [36]. Thus, finding things that make people feel more competent, such as conforming to the mask-wearing attitudes of those in their ingroup, may provide people with a sense of self-verification and positive feedback that their behaviors and beliefs are correct. Additionally, people tend to notice or pay greater attention to factors that confirm their beliefs [62,63], such as potentially focusing more on the minor discomforts associated with mask wearing (e.g., facial temperature, breathing resistance, headaches, etc.) to provide validation and feelings of competence, which may then serve to enhance perceptions of discomfort. Thus, the perceived discomforts and negative attitudes associated with wearing masks during the COVID-19 pandemic may be at least partially explained by attempts to satisfy the three basic psychological needs of autonomy, relatedness, and competence, as opposed to any real physiological drawbacks or discomforts. 

## 7. Conclusions

In otherwise healthy individuals, wearing masks, even for an extended period of time, does not produce any clinically relevant changes in circulating O_2_ or CO_2_ concentrations, and does not seem to impact tidal volume or respiratory rate. However, wearing a mask does produce a small increase in breathing resistance caused by the mask material filtering particles and aerosols in the air and any moisture that is trapped in the mask material. One consistently documented negative impact of wearing a mask for a long period of time is an increase in the development of headaches in people with a history of headaches. Yet overall, the virus reduction and therefore potentially life-saving benefits of wearing face masks seem to outweigh the discomforts. The impact of mask wearing on basic psychological needs (autonomy, relatedness, and competence) is likely a contributor to the controversy associated with wearing masks during the COVID-19 pandemic in the United States; however, future research is needed to empirically test this theoretical evidence. It is important to note that this commentary discussed some overarching psychological factors that may contribute to mask-wearing attitudes and behaviors. However, several other factors may also contribute to the decision to wear a mask and would warrant investigation in future research. Some examples include altruism, self-efficacy, risk assessment, need for control or certainty, self-serving bias, perceptions of fairness, ability to engage in hot vs. cold cognition, short-term vs. long-term orientation, restraint vs. indulgence, trust in science, socioeconomic status, education level, personal experience, and other personality or physiological individual differences.

The current commentary focused on the impact of wearing a face mask from an individual-level. It is important to acknowledge that there may be broader associations and implications of wearing a face mask not discussed. For example, there may be meso-level impacts (i.e., medium systems, such as organizational, ethnic, and community), and macro-level impacts as well (i.e., large systems, such as a national economy). To our knowledge, there is currently no research that examines face mask wearing and the impact of more meso-and macro level systems on the current COVID-19 pandemic. 

Future research is needed to determine the impacts of wearing cloth masks during everyday activities, higher intensity physical activities, and in special populations, as the COVID-19 pandemic may require this necessity for a longer period of time. Specifically, randomized controlled trials investigating different mask types (different materials, fits, brands) during a variety of activities and measuring the physiological impacts and/or the protective effects for the wearer or others would be helpful for the public. More quality studies from scientists will help support public health officials when they are encouraging or mandating mask wearing. More data continues to emerge supporting the use of masks; for example, one recent study examined the different sources of variation among over 200 countries in per-capita mortality due to COVID-19, and reported that duration of mask wearing by the public was negatively associated with mortality [64]. Thus, masks may be here to stay for the foreseeable future and may not just have application for COVID-19, but for severe flu seasons and for potential future respiratory epidemics/pandemics. Future research should also focus on how we can encourage the better adoption of masks usage and how we can decrease perceptions of discomfort, all while focusing on the global task at hand: controlling and eliminating COVID-19. Future research will need to shed light on effective public health programs (including health education programs) that can maximize mask usage and compliance of mask wearing and explore the physiological and psychological impacts of these programs.
